# Advancing Cervical Cancer Prevention Initiatives in Resource-Constrained Settings: Insights from the Cervical Cancer Prevention Program in Zambia

**DOI:** 10.1371/journal.pmed.1001032

**Published:** 2011-05-17

**Authors:** Mulindi H. Mwanahamuntu, Vikrant V. Sahasrabuddhe, Sharon Kapambwe, Krista S. Pfaendler, Carla Chibwesha, Gracilia Mkumba, Victor Mudenda, Michael L. Hicks, Sten H. Vermund, Jeffrey S. A. Stringer, Groesbeck P. Parham

**Affiliations:** 1 Centre for Infectious Disease Research in Zambia, Lusaka, Zambia; 2 University Teaching Hospital, Lusaka, Zambia; 3 Vanderbilt University, Nashville, Tennessee, United States of America; 4 University of Cincinnati, Cincinnati, Ohio, United States of America; 5 University of Alabama at Birmingham, Birmingham, Alabama, United States of America; 6 Michigan Cancer Institute, Pontiac, Michigan, United States of America

## Abstract

Groesbeck Parham and colleagues describe their Cervical Cancer Prevention Program in Zambia, which has provided services to over 58,000 women over the past five years, and share lessons learned from the program's implementation and integration with existing HIV/AIDS programs.

**Summary Points**

- Invasive cervical cancer is a leading cause of cancer-related death and morbidity among women in the developing world.
- Screening coverage rates are very low in developing countries despite there being proven, simple, “screen and treat” approaches for cervical cancer prevention.
- In 2006 we initiated a partnership with the public health system in Zambia and created the Cervical Cancer Prevention Program in Zambia (CCPPZ), targeting the highest risk HIV-infected women, and have provided services to over 58,000 women (regardless of HIV status) over the past 5 years.
- We have demonstrated a strategy for using the availability, momentum, and capacity-building efforts of vertical HIV/AIDS care and treatment programs to implement a setting-appropriate protocol for cervical cancer prevention within public health infrastructures.
- We report our lessons learned to help other cervical cancer prevention initiatives succeed in the developing world and to avoid additional burdens on health systems.

## The Challenge of Implementing Cervical Cancer Prevention in the Developing World

**Figure 1:**
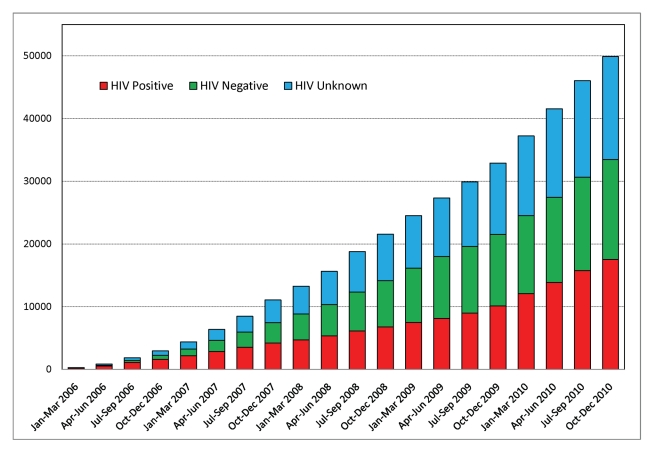
Cumulative enrollment graph of women accessing services in the Cervical Cancer Prevention Program in Zambia between 2006 and 2010.

**Figure 2:**
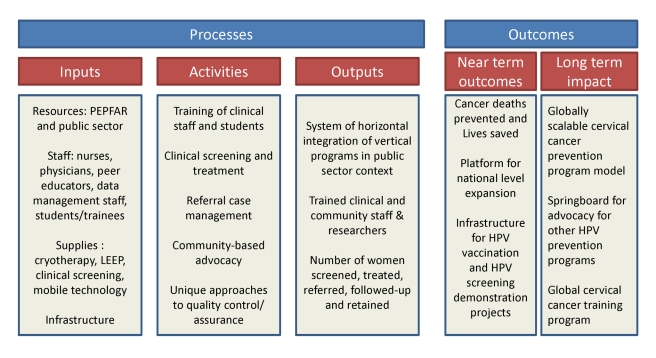
Program evaluation indicators of the Cervical Cancer Prevention Program in Zambia.

Invasive cervical cancer (ICC) is a leading cause of cancer-related mortality and morbidity among women in the developing world [1]. Numerous demonstration projects have proven the efficacy of simplified “screen and treat” approaches (such as visual inspection with acetic acid [VIA] and immediate cryotherapy) for cervical cancer prevention in low income countries [2]–[5]. Yet, rarely have they been adopted and scaled up by governments in such settings, and as a result rates of screening coverage in the developing world remain low [6]. In Zambia, which has the world's second highest annual cervical cancer incidence and mortality rates [1], we initiated a unique partnership for providing cervical cancer prevention services within the public sector health care system. The Cervical Cancer Prevention Program in Zambia (CCPPZ), launched in 2006 and initially targeting the highest risk HIV-infected women, has cumulatively provided services to over 58,000 women (regardless of HIV status) over the past 5 years [7],[8] (Figure 1). Our previous reports have described the disease burden [9],[10],[11], program implementation experience [7], telecommunications matrix utilized for distance consultation and quality assurance [8], outcomes of a referral system for women ineligible for treatment with cryotherapy [12], common myths and misconceptions surrounding cervical cancer [13], and an analysis of outcomes and effectiveness of screening among women with HIV [11]. In this article, we discuss the unique attributes of the program's successful implementation, including its adoptability to the conditions of a resource-constrained environment, integration with infrastructure of an HIV/AIDS program, use of innovative quality assurance measures, and the potential for sustainability and collateral impact (Figure 2). The global “call to action” for cancer control in low income countries is emerging [14], yet funding for stand-alone cancer prevention initiatives is virtually non-existent. Our experience may provide insights and guidance for advancing cancer prevention initiatives in resource-constrained settings.

## The Cervical Cancer Prevention Program in Zambia (CCPPZ)

CCPPZ is a unique partnership between Zambian and United States partner institutions from the academic (University Teaching Hospital [UTH] in Lusaka and University of Alabama at Birmingham [UAB]), research (Centre for Infectious Disease Research in Zambia [CIDRZ]), and governmental (Zambian Ministry of Health) sectors. Prevention efforts have been rapidly scaled up, taking advantage of a historical circumstance of increased attention and funding in the HIV/AIDS sector through the US President's Emergency Plan for AIDS Relief (PEPFAR) program [15]. Trained nurses form the backbone of the program, providing free personalized clinical screening examinations for all eligible women who attend the clinic [7],[11]. VIA aided by a low-cost digital camera adaptation (“digital cervicography”) is used to provide results immediately [11]. Decisions about offering immediate cryotherapy (“screen-and-treat”) or referral are made independently by nurses in most cases, or aided by telemedicine consultations with local doctors [11]. VIA-negative women are advised to follow-up after 1 year, VIA-positive and cryotherapy-eligible women are treated by the nurses, and those needing referral are advised to attend the Gynecologic Cancer Prevention Unit in the UTH [12]. Administrative support is provided by CIDRZ, while program operations are integrated with the Zambian Ministry of Health's public sector health care infrastructure [7]. From 2006 to March 2011, over 58,000 women, regardless of HIV status, have been screened in this program. Of the initial 21,010 women who were provided services in the program (31% of whom had HIV), 38% were VIA positive, of whom 62% were eligible for cryotherapy and 38% were referred for histologic evaluation. Forty-nine percent of patients referred for histologic evaluation complied, and pathology results revealed benign abnormalities in 8%, CIN1 in 28%, CIN2/3 in 26%, and ICC in 19% (of which 55% were early stage).

## Low-Tech Yet Scalable Prevention Intervention Modality

The dearth of adequately trained cytotechnologists and pathologists, and the requirement for multiple patient visits, preclude successful utilization of cervical cytology (“Pap smears”) in most of the developing world [5]. Testing for cervical human papillomavirus (HPV) is a highly accurate screening approach, [16],[17] but the high cost of current commercially available HPV testing technology makes routine implementation in low income countries impractical, if not impossible [18]. VIA linked to cryotherapy has unique strengths (it can be done by mid-level health care providers, and has immediate results that allow linking screening and treatment in the same visit), but also antecedent weaknesses (higher potential for false-positive results, and limitations in ensuring quality assurance [5]). Nonetheless, VIA remains the only available option for a setting like Zambia. We enhanced its application through the use of digital photography (cervicography) as a routine adjunct to ensure quality as well as help with patient education and electronic record keeping, and rapid distance consultation with the limited number of available physician experts, when necessary [8]. Our model facilitated program scale-up, despite limited resources. In fact, our experience suggests that while the accuracy of a screening test is an important attribute, it is only a small part of the equation for mitigating cervical cancer. Ultimately, the success of cervical cancer prevention interventions is largely determined by how screening and treatment activities are integrated within the routine health care system to ensure maximal impact, an enduring goal of our program.

## Horizontal Integration with Donor-Funded HIV/AIDS Care and Treatment Programs

The increasing expansion, over the past decade, of bilateral (e.g., PEPFAR) and multilateral (e.g., Global Fund) donor funding for HIV/AIDS programs has transformed health care delivery in resource limited settings, particularly in sub-Saharan Africa [19]. With support from PEPFAR and private donations, CCPPZ's cervical cancer prevention clinics are co-located within public health clinics offering HIV/AIDS care and treatment. Manifold advantages of this strategy include resource and infrastructure sharing, availability of a wider range of women's health services for HIV-infected/at-risk women, and opportunities for referral between the clinic systems and maximization of participation in both programs. Yet, the stigma associated with HIV/AIDS continues to be a challenge in Zambia, which drove our decision to keep service provisions and the identities of the cervical cancer and HIV/AIDS care programs distinct, albeit in the same premises. Women attending cervical cancer screening clinics with unknown HIV status are offered HIV testing within the confidential environment of the screening rooms. Indeed, cervical cancer itself is stigmatized, and anxiety and fear often keep women from getting screened [13]. In response, the program is branded as a “Cervical Health” program, which deemphasizes cancer while denoting a positive (“health”) connotation to the effort.

## Innovations Using Information Technology

One of the most enabling and transforming aspects of the program involves utilizing information technology tools for enhancing the program's operations and enabling quality assurance. Telemedicine consultation through the “electronic cervical cancer control” model [8] is a mobile health application that has enhanced clinical care services and improved quality assurance of nurse-led VIA. The use of community volunteers as patient-tracking officers, taking advantage of the wide usage of cell phone–based communication among the patient population, is now promoting adherence to the already minimized follow-up visit requirements. The use of point-of-care online data entry (undertaken by nurses and their community volunteer assistants), and a centralized Web-based patient clinical and laboratory records management system are enhancing program monitoring and outcomes evaluation.

## Collateral Impact and the Promise of Sustainability

**Figure 3:**
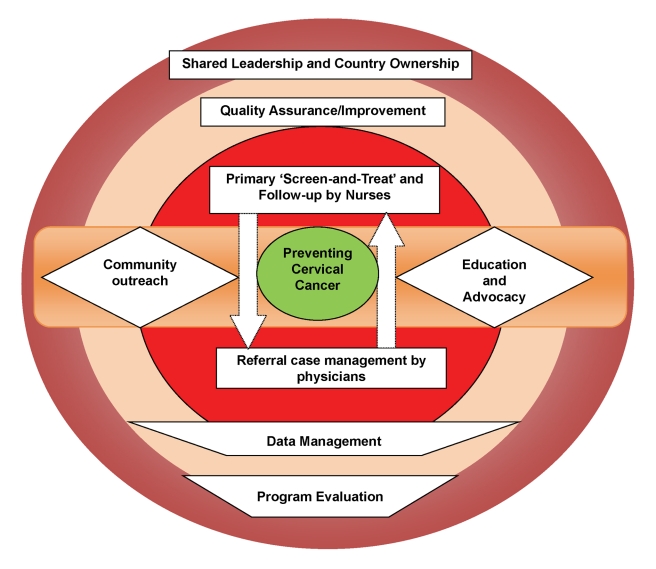
Conceptual Framework guiding the program development and operations of the Cervical Cancer Prevention Program in Zambia.

The program is, by design, integrated with the operations of the public health system run and operated by the Zambian Ministry of Health, a model that has helped its transition from being a foreign-funded/led project to a locally/nationally “owned” program. Program nurses are recruited from the public sector health care system, with add-on financial incentives to facilitate retention. The program also employs community-based volunteers to serve as “peer educators” to facilitate community awareness, counteract misconceptions and myths, and serve patient support functions [13]. All referral services are located within UTH, the only state-run tertiary hospital in Zambia, which permits medical and surgical services (including loop electrosurgical excision procedures [LEEP], hysterectomy, radiation, and chemotherapy) at low-to-no cost to patients. The need for coordination of various administrative, clinical, and laboratory (referral-level) services are achieved through locally employed Zambian nationals, with technical assistance from expatriate American clinicians and administrators living within the country, in a “shared leadership” model (Figure 3). The motto of the program (“Every woman has the right to live a life free from cervical cancer”) encapsulates the rights-based approach to women's health, designed to promote community-based acceptance of this program. The program's infrastructure has facilitated the development of a linked clinical, epidemiological, and translational research platform supported by numerous extramural research and training grants and contracts. Opportunities abound for engaging Zambian and foreign clinical and research trainees. The program's successful expansion in the Lusaka area has leveraged political will for nationwide expansion, as well as eventual roll out of HPV vaccination and HPV-based screening [20] as the logical next steps in its evolution (Figure 2). As with any expanding program, unique challenges may arise with future geographic expansion, yet we believe that the lessons learnt during our initial years as well as the improved awareness that the program has engendered, will likely generate unique solutions.

## Conclusion

For cancer prevention initiatives to succeed in the developing world, programs must avoid placing additional burdens on health systems already stretched thin due to competing priorities. We have demonstrated a strategy for using the availability, momentum, and capacity-building efforts of vertical HIV/AIDS care and treatment programs to implement other disease-specific initiatives such as cervical cancer prevention [19]. By integrating a setting-appropriate protocol for cervical cancer prevention into public health infrastructures, and promoting shared leadership with government ownership, our program has not just saved lives [11], but has also established a new solution for routine prevention intervention in resource-constrained environments.

## Abbreviations

CCPPZ: Cervical Cancer Prevention Program in Zambia
CIDRZ: Centre for Infectious Disease Research in Zambia
HPV: human papillomavirus
ICC: invasive cervical cancer
PEPFAR: US President's Emergency Plan for AIDS Relief
UTH: University Teaching Hospital
VIA: visual inspection with acetic acid
